# Late Migration of an Adjustable-Loop Cortical Suspension Device in Anterior Cruciate Ligament Reconstruction

**DOI:** 10.1155/2019/1061385

**Published:** 2019-08-19

**Authors:** Brendan A. Williams, Jorge Gil, Kevin W. Farmer

**Affiliations:** ^1^Department of Orthopaedic Surgery, Children's Hospital of Philadelphia, Philadelphia, PA, USA; ^2^Department of Orthopaedics & Rehabilitation, University of Florida, Gainesville, FL, USA

## Abstract

A unique case of late migration of an adjustable-loop femoral fixation button utilized during anterior cruciate ligament (ACL) reconstruction is presented. Imaging and physical examinations during the patient's postoperative course were unremarkable for graft or hardware failure. Two years postoperatively, symptomatic hardware migration occurred requiring arthroscopic removal. To our knowledge, this is the first reported case of late migration of an adjustable-loop femoral fixation button in ACL reconstruction. This case highlights that late loosening and migration of adjustable-loop femoral fixation devices in ACL reconstruction can occur despite demonstrated postoperative radiographic and clinical stability. Surgeons utilizing this fixation device should be aware of this potential complication to avoid delayed recognition and patient morbidity.

## 1. Introduction

Methods of femoral fixation in ACL reconstruction surgery are widely discussed in the literature. The cortical suspension technique [1] is a popular option with a variety of adjustable-loop and fixed-loop devices available on the market. Techniques have been described that allow direct arthroscopic visualization of the fixation button during application in the femur [2] helping to avoid some of the commonly cited complications seen utilizing other cortical suspension devices including soft tissue interposition and hardware migration [3]. Adjustable-loop devices are additionally desirable due to ease of insertion, allowance of femoral tunnel graft fill, and knotless security. The adjustable-loop ACL TightRope RT (Arthrex, Naples, FL) is routinely utilized for ACL reconstructions performed at our institution for these reasons. Roughly 800 ACL reconstructions have been performed at our center using this technique for femoral fixation by two high-volume, fellowship-trained orthopaedic sports surgeons without known suture-button migration. In this study, we report a rare case of late, symptomatic migration of an Arthrex TightRope RT button requiring reoperation for arthroscopic removal two years postoperatively.

## 2. Case Report

In 2013, a 24-year-old healthy female presented to our clinic 4 months after sustaining a martial arts twisting injury to her left knee. Continued pain and mechanical symptoms despite activity rest and conservative measures prompted an MRI study by her primary care physician and subsequent referral to our care based on the imaging findings of an ACL tear. The patient reported persistent instability and locking of her knee occurring with all activities and had failed trials of bracing, NSAIDs, and icing. She was unable to return to sport or her desired level of activity, and she desired surgical intervention.

Surgical intervention was recommended given her age and activity level. The patient elected to proceed with ACL reconstruction with allograft after review of the surgical procedure and available graft options. Preoperative physical exam demonstrated a positive Lachman exam. Maximum knee joint arthrometer testing (KT-2000 MEDmetric Corp, San Diego, CA) was 7 mm on the right and 13 mm on the left. The patient underwent arthroscopically assisted ACL reconstruction with a 9 mm tibialis anterior allograft. Fixation included a 10 × 28 mm Arthrex BioComposite interference screw distally with an ACL TightRope RT for femoral fixation using outside-in tunnel retrodrilling technique. Button passage and confirmation of flip onto the lateral femoral cortex was visualized via the anteromedial portal through the 3.5 mm lateral cortical tunnel. Intraoperative findings were significant for ACL tear only without additional pathology noted. The procedure completed without complication.

Rehab consisted of knee immobilization and nonweight-bearing precautions immediately postoperatively. Range of motion was initiated on postoperative day one. Weight-bearing was advanced to as tolerated on postoperative day one. Jogging was initiated at three months. She completed postoperative rehabilitation without issue and returned to her desired level of activity by six months. Maximum KT-2000 testing at 6 months was 8 mm on the right and 9 mm on the left. Biodex testing at this time demonstrated 13.9% extension and 17.3% flexion deficit at 180 degrees/second.

The patient had an unremarkable immediate postoperative course marred only by a few brief periods of activity-related knee pain resolving with conservative measures including a single pes anserine steroid injection one year postoperatively for pes bursitis.

**Figure 1:**
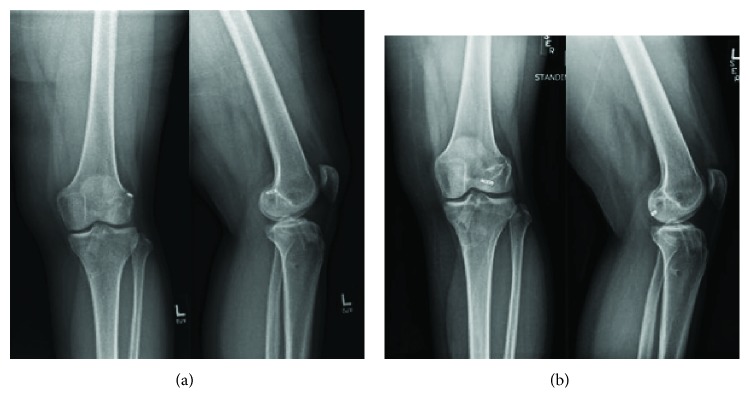
(a) Eighteen-month postoperative radiographs of the left knee performed as a screening examination due to pain following minor knee trauma. AP: lateral and notch views demonstrate expected postoperative changes from ACL reconstruction with well-positioned femoral tightrope button. (b) Two-year postoperative radiographs of the left knee performed due to new onset of symptoms in the posterior knee. There is evidence of interval migration of the TightRope button into the posterolateral joint space.

Eighteen months postoperatively, the patient presented to the clinic due to acutely worsening anteromedial knee pain after being kicked in the leg during martial arts practice. Her pain was worst with knee extension, but she denied mechanical symptoms or instability. Physical exam at this time was unremarkable and radiographs (Figure 1(a)) demonstrated no evidence of complication of her reconstruction as compared with other imaging during her postoperative course. These issues subsequently resolved with conservative management strategies.

Two years postoperatively, the patient returned for worsening posterior knee pain. She reported a pinching sensation in the back of her knee that was most prominent at full extension and while walking. She had already begun to use crutches for ambulation due to these symptoms at the time of her visit. Radiographs at this time demonstrated a migrated TightRope button (Figure 1(b)). Physical exam revealed no ligamentous laxity and was remarkable only for pinching pain throughout passive range of motion. An MRI was obtained, and the migrated hardware was again visualized in the posterior knee joint, along with an intact-appearing ACL graft.

**Figure 2:**
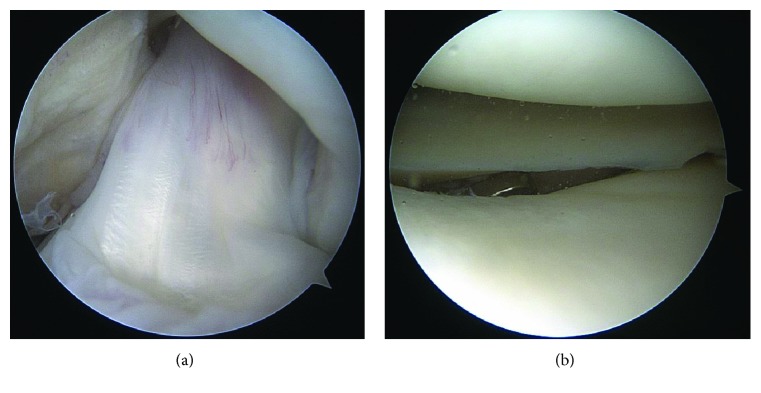
(a) Arthroscopic notch view visualized through the anterolateral portal demonstrating a well-vascularized ACL graft. (b) Arthroscopic view of the lateral compartment visualized through the anteromedial portal demonstrating the migrated TightRope button under the posterior horn of lateral meniscus.

The patient was counseled on arthroscopic exploration and removal of the migrated hardware. Diagnostic arthroscopy revealed a stable-appearing ACL graft with probing that appeared well vascularized (Figure 2(a)). The TightRope button was located in the posterolateral knee, under the posterior horn of the lateral meniscus (Figure 2(b)). There was no TightRope suture attached to the button, and the suture was not visualized in the knee. The button was retrieved uneventfully. Postoperatively, the patient's pain and motion limitations associated with the displaced button resolved.

## 3. Discussion

Cortical suspension exists as a well-established and reliable method of femoral graft fixation in ACL reconstruction surgery. Though complications are rare, the most commonly cited failures include tunnel widening, intra-tunnel fixation, and soft tissue interposition [3]. Each of these can result in acute or late hardware migration with or without graft failure. In a review of patients undergoing anatomic double-bundle ACL reconstruction with use of a fixed-loop EndoButton CL (Smith & Nephew Endoscopy, Andover, MA), Mae et al. found a high incidence of both soft tissue interposition (25%) and EndoButton migration (35%) noted on postoperative radiographs [3]. Similarly, Taketomi et al. identified a high prevalence of postoperative migration (9%) in addition to a significant rate of hardware rotation (56%) [4]. Neither study, however, appeared to show a significant effect on clinical or patient-reported outcomes, and there were no noted migrations of the EndoButton into the knee joint or migrations requiring reoperation in either cohort.

Graft fixation with adjustable-loop cortical suspension remains a relatively new technique [1] with few long-term studies examining potential complications with this fixation device. Although recent biomechanical studies have raised concerns about loop lengthening and fixation strength of adjustable-loop constructs as compared to fixed-loop designs [5–8], others suggest these biomechanical differences may be clinically insignificant to postoperative knee stability [9–11]. In our personal experience, we have not identified graft failures or required reoperations due to loop lengthening or hardware migration with the adjustable-loop construct.

It has been hypothesized that due to the relative anatomical soft tissue complexity of the distal femur, the success of a cortical suspension may be associated to the location of device placement [12]. Graft and button placement about the distal femur is also impacted by the method of femoral drilling, which in our case was performed via outside-in retrodrilling. A recent study by Uchida et al. found that 54.3% of cortical buttons located posterior to the femoral lateral supracondylar line migrated while only 15.1% of cortical buttons located anterior to this line changed position [12]. This migration is thought to be secondary to increased posterior femoral soft tissue—increasing the chance of soft tissue necrosis and/or loosening due to repetitive tissue contraction [12].

Though local migration with cortical suspension devices may occur, distant, and symptomatic hardware migration is exceedingly rare. A literature search within the English-language literature yielded two case reports of late cortical fixation device migration into the knee joint [13, 14]. In both cases, similar fixed-loop fixation devices were utilized, with knots tied for final fixation. One report questioned whether proximity of the fixation button to the femoral groove contributed to the late failure [14]. The second report attributed knot loosening to intra-articular fluid effect [13]. To our knowledge, late migration of an adjustable-loop femoral button has not been previously reported in the literature. In our case, arthroscopy for hardware retrieval was effective for resolving the patient's symptoms. The button was intact and no suture was identified within the joint or attached to the button at retrieval. This suggests that the connection of the button and sutures had failed, perhaps via micromotion of the button along the lateral cortex of the femur during knee motion. However, because no graft-related issues were identified during the patient's postoperative course, the device still allowed adequate time for graft healing prior to button loosening and migration. Surgeons utilizing this fixation device should be aware of this potential complication to avoid delayed recognition and associated patient morbidity.
